# Supplementary material for: Deficits in Mitochondrial Spare Respiratory Capacity Contribute to the Neuropsychological Changes of Alzheimer’s Disease
Source: J Pers Med. 2020 Apr 29;10(2):32. doi: 10.3390/jpm10020032 (PMC7354560; doi:10.3390/jpm10020032)
Supplement: Supplementary file 1 [file jpm-10-00032-s001.pdf]

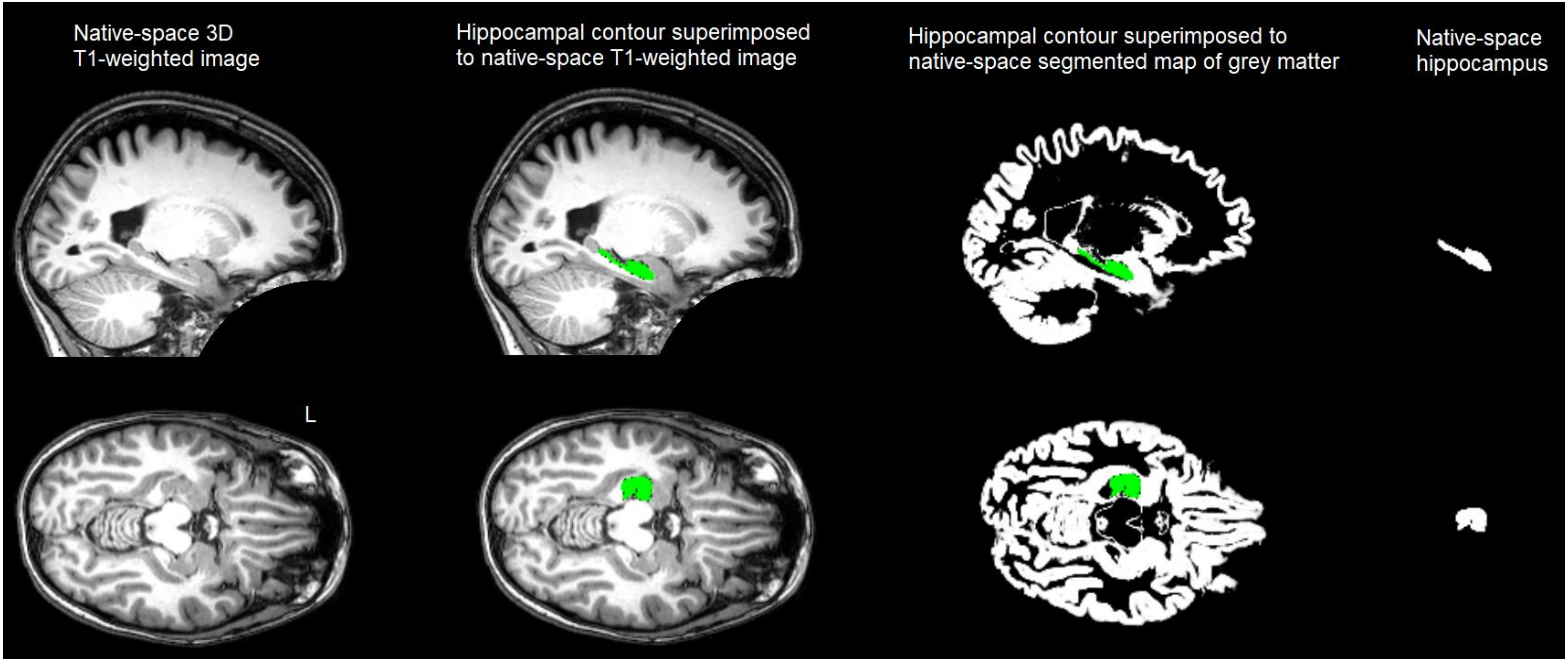
Native-space 3D T1-weighted image
Hippocampal contour superimposed to native-space T1-weighted image
Hippocampal contour superimposed to native-space segmented map of grey matter
Native-space hippocampus
L
